# 4-(4-Fluoro­phen­yl)-6-methyl­amino-5-nitro-2-phenyl-4*H*-pyran-3-carbo­nitrile

**DOI:** 10.1107/S1600536813009008

**Published:** 2013-04-10

**Authors:** R. Vishnupriya, J. Suresh, S. Sivakumar, R. Ranjith Kumar, P. L. Nilantha Lakshman

**Affiliations:** a Department of Physics, The Madura College, Madurai 625 011, India; b Department of Organic Chemistry, School of Chemistry, Madurai Kamaraj University, Madurai 625 021, India; c Department of Food Science and Technology, University of Ruhuna, Mapalana, Kamburupitiya 81100, Sri Lanka

## Abstract

In the title compound, C_19_H_14_FN_3_O_3_, the central pyran ring adopts a boat conformation with the O atom and the quaternary C atom diagonally opposite displaced by 0.068 (1) and 0.075 (1) Å, respectively, above the mean plane defined by the other four ring atoms. The co-planar atoms of the pyran ring and the fluoro­phenyl ring are nearly perpendicular, as evidenced by the dihedral angle of 87.11 (1)°. The amine group forms an intra­molecular N—H⋯O(nitro) hydrogen bond. In the crystal, mol­ecules are linked into parallel chains along [100] by weak N—H⋯N and C—H⋯N(nitro) hydrogen bonds, generating *C*(8) and *C*(9) graph-set motifs, respectively.

## Related literature

For the biological activity of substituted pyran derivatives, see: Lokaj *et al.* (1990 ▶); Marco *et al.* (1993 ▶). Some 4*H*-pyran derivatives are potential bioactive compounds and can be used as calcium antagonists, see: Suárez *et al.* (2002 ▶). For hydrogen-bonding graph-set motifs, see: Bernstein *et al.* (1995 ▶). For ring conformation analysis, see: Cremer & Pople (1975 ▶). The title compound and some related compounds are widely used as organic inter­mediates in organic chemistry (Liang *et al.*, 2009 ▶). For related structures, see: Nesterov *et al.* (2004 ▶); Nesterov & Viltchinskaia (2001 ▶). For a description of the Cambridge Structural Database, see: Allen (2002 ▶). For standard bond lengths, see: Allen *et al.* (1987 ▶).
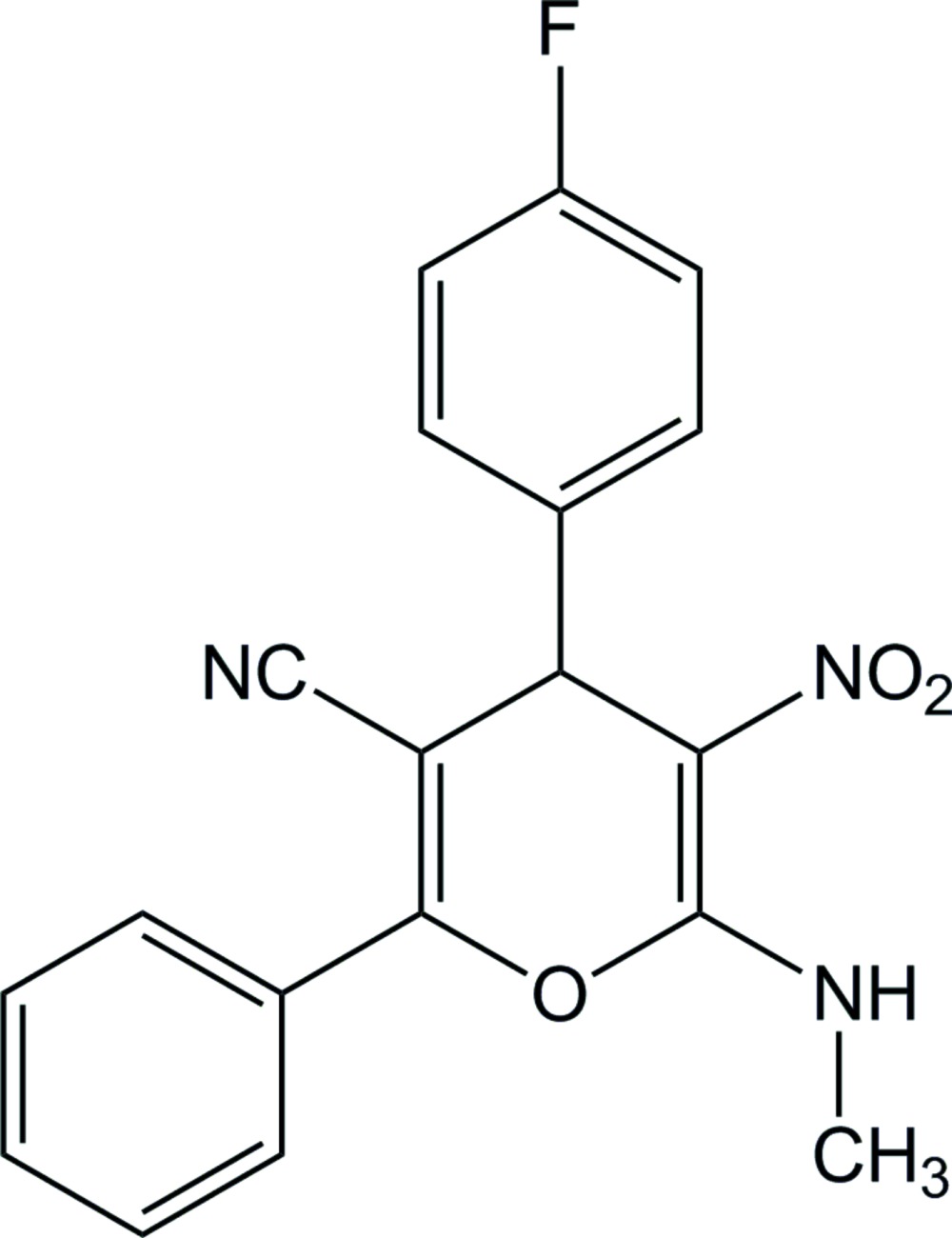


## Experimental

### 

#### Crystal data

C_19_H_14_FN_3_O_3_
*M* *_r_* = 351.33
Triclinic, 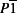

*a* = 9.3898 (3) Å
*b* = 9.9752 (3) Å
*c* = 11.1324 (3) Å
α = 98.765 (1)°
β = 113.991 (1)°
γ = 109.520 (1)°
*V* = 846.09 (4) Å^3^
*Z* = 2
Mo *K*α radiation
μ = 0.10 mm^−1^
*T* = 293 K
0.23 × 0.20 × 0.19 mm

#### Data collection

Bruker Kappa APEXII diffractometer
Absorption correction: multi-scan (*SADABS*; Sheldrick, 1996 ▶) *T*_min_ = 0.967, *T*_max_ = 0.974
16948 measured reflections
3680 independent reflections
2993 reflections with *I* > 2σ(*I*)
*R*_int_ = 0.026

#### Refinement

*R*[*F* ^2^ > 2σ(*F* ^2^)] = 0.040
*wR*(*F* ^2^) = 0.118
*S* = 1.06
3680 reflections
236 parameters
H-atom parameters constrained
Δρ_max_ = 0.24 e Å^−3^
Δρ_min_ = −0.22 e Å^−3^

### 

Data collection: *APEX2* (Bruker, 2004 ▶); cell refinement: *SAINT* (Bruker, 2004 ▶); data reduction: *SAINT*; program(s) used to solve structure: *SHELXS97* (Sheldrick, 2008 ▶); program(s) used to refine structure: *SHELXL97* (Sheldrick, 2008 ▶); molecular graphics: *PLATON* (Spek, 2009 ▶); software used to prepare material for publication: *SHELXL97*.

## Supplementary Material

Crystal structure: contains datablock(s) global, I. DOI: 10.1107/S1600536813009008/bh2475sup1.cif

Structure factors: contains datablock(s) I. DOI: 10.1107/S1600536813009008/bh2475Isup2.hkl

Supplementary material file. DOI: 10.1107/S1600536813009008/bh2475Isup3.cml

Additional supplementary materials:  crystallographic information; 3D view; checkCIF report

## Figures and Tables

**Table 1:** Hydrogen-bond geometry (Å, °)

*D*—H⋯*A*	*D*—H	H⋯*A*	*D*⋯*A*	*D*—H⋯*A*
N2—H2⋯O2	0.86	1.99	2.6089 (16)	128
N2—H2⋯N3^i^	0.86	2.30	2.9811 (17)	136
C6—H6*A*⋯N3^i^	0.96	2.60	3.222 (2)	123

Symmetry code: (i) 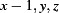.

**Fig. 1.**
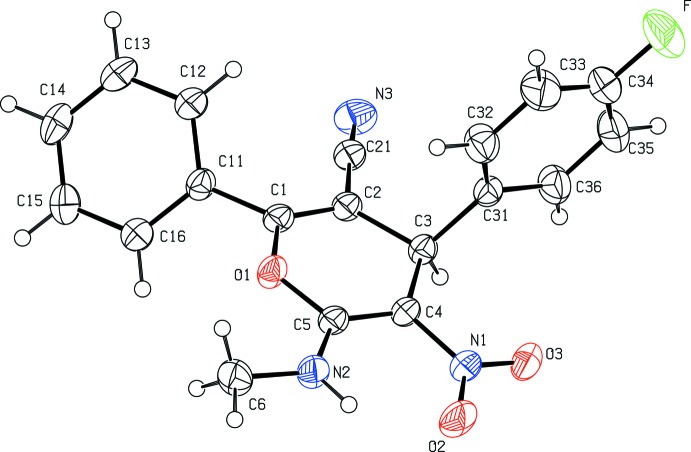
The molecular structure of the title molecule, showing 40% probability displacement ellipsoids for non-H atoms.

**Fig. 2.**
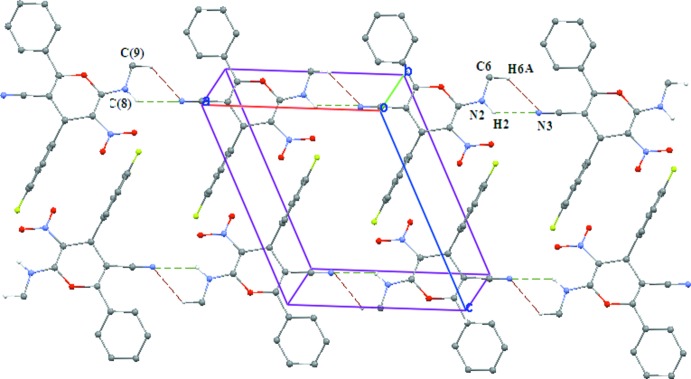
Packing diagram showing the chain motifs C(8) and C(9) along the [100] direction.

## supplementary crystallographic information

### Comment

The title compound and some related compounds are widely used as organic intermediates in organic chemistry (Liang *et al.*, 2009). Much interest has recently been paid to the design of polyfunctionalized substituted pyran derivatives, owing to their wide range of biological activities (Lokaj *et al.*, 1990; Marco *et al.*, 1993). Some 4*H*-pyran derivatives are potential bioactive compounds and can be used as calcium antagonists (Suárez *et al.*, 2002). Thus, there has been a growing interest in the structures of 4*H*-pyran derivatives. The high biologically active value of these compounds in conjunction with our research interests prompted us to synthesize and report the X-ray study of the title compound.

In the title compound (C_19_H_14_FN_3_O_3_, Fig. 1) the six-membered central pyran ring adopts a boat conformation as evidenced by the puckering parameters q_2_ = 0.0826 (12) Å, θ = 88.18 (4)°, φ = 127.06 (4)° (Cremer & Pople, 1975). The dihedral angle between the pseudo-axial aryl substituent and the flat part of the pyran ring is 87.11 (1)°. There is conjugation between the donor (NH) and the acceptor (CN) groups *via* the C4═C5 double bond, as found in other related compounds (Nesterov *et al.*, 2001, 2004). Thus, the C5—N2 distance is 1.3130 (17) Å, which is shorter than the average conjugated C—N single bond, 1.370 (1) Å, found in the Cambridge Structural Database (Allen, 2002). In contrast, the C4═C5 bond is elongated in comparison with the C1═C2 bond and the standard value (Allen *et al.*, 1987). The C4—N1 distance, 1.3855 (17) Å, is considerably shorter than usual C—NO_2_ distance (1.468 Å, Allen *et al.*, 1987) and the N1—O2 distance, 1.2558 (16) Å, is longer than the standard value (Allen *et al.*, 1987). The dihedral angle between the flat part of the pyran ring and the phneyl ring at C1 is 49.22 (2)°. The phenyl and the fluorophenyl rings are substituting the pyran ring in a (–)-*syn*-clinal conformation, with torsion angles C2—C1—C11—C12 and C4—C3—C31—C32 of -51.4 (2)° and -60.76 (17)° respectively. The nitro group is bonded to the pyran ring at C4 with the torsion angle C5—C4—N1—O2 of -7.09 (3)°, indicating a (–)-*syn*-periplanar conformation for this group.

In the crystal structure, the molecules are linked together, to form an infinite one dimensional chain along [100], through intermolecular N2—H2···N3 and C6—H6A···N3 hydrogen bonds, generating graph set motifs *C*(8) and *C*(9) respectively (Fig. 2; Bernstein *et al.*, 1995). In addition, there is a N—H···O intramolecular interaction which stabilizes the molecular conformation.

### Experimental

A mixture of benzoylacetonitrile (1.0 mmol), 4-fluoroaldehyde (1.0 mmol), Et_3_N (1.0 mmol) and 10 ml EtOH were taken in 50 ml round bottom flask. The reaction mixture was stirred at room temperature for 5–10 min. Then *N*-methyl-1-(methylthio)-2-nitroethenamine was added into the reaction mixture and the system refluxed at 80°C. The consumption of starting material was monitored by TLC. After 90 min., the solid product was filtered and washed with diethyl ether (5 ml) and dried under vacuum condition to afford the pure product. Melting point: 210°C; Yield: 94%

### Refinement

H atoms were placed at calculated positions and allowed to ride on their carrier atoms with C—H = 0.93–0.98 Å, N—H = 0.86 Å. *U*_iso_ =1.2*U*_eq_(C,N) for NH and CH groups and *U*_iso_ = 1.5*U*_eq_(C6) for the methyl group.

### Crystal data

**Table:**

C_19_H_14_FN_3_O_3_	*Z* = 2
*M**_r_* = 351.33	*F*(000) = 364
Triclinic, *P*1	*D*_x_ = 1.379 Mg m^−^^3^
Hall symbol: -P 1	Melting point: 483 K
*a* = 9.3898 (3) Å	Mo *K*α radiation, λ = 0.71073 Å
*b* = 9.9752 (3) Å	Cell parameters from 2000 reflections
*c* = 11.1324 (3) Å	θ = 2–27°
α = 98.765 (1)°	µ = 0.10 mm^−^^1^
β = 113.991 (1)°	*T* = 293 K
γ = 109.520 (1)°	Block, colourless
*V* = 846.09 (4) Å^3^	0.23 × 0.20 × 0.19 mm

### Data collection

**Table:**

Bruker Kappa APEXII diffractometer	3680 independent reflections
Radiation source: fine-focus sealed tube	2993 reflections with *I* > 2σ(*I*)
Graphite monochromator	*R*_int_ = 0.026
Detector resolution: 0 pixels mm^-1^	θ_max_ = 27.0°, θ_min_ = 2.1°
ω and φ scans	*h* = −11→11
Absorption correction: multi-scan (*SADABS*; Sheldrick, 1996)	*k* = −11→12
*T*_min_ = 0.967, *T*_max_ = 0.974	*l* = −14→14
16948 measured reflections	

### Refinement

**Table:**

Refinement on *F*^2^	Secondary atom site location: difference Fourier map
Least-squares matrix: full	Hydrogen site location: inferred from neighbouring sites
*R*[*F*^2^ > 2σ(*F*^2^)] = 0.040	H-atom parameters constrained
*wR*(*F*^2^) = 0.118	*w* = 1/[σ^2^(*F*_o_^2^) + (0.054*P*)^2^ + 0.2234*P*] where *P* = (*F*_o_^2^ + 2*F*_c_^2^)/3
*S* = 1.06	(Δ/σ)_max_ < 0.001
3680 reflections	Δρ_max_ = 0.24 e Å^−^^3^
236 parameters	Δρ_min_ = −0.22 e Å^−^^3^
0 restraints	Extinction correction: *SHELXL97* (Sheldrick, 2008), Fc^*^=kFc[1+0.001xFc^2^λ^3^/sin(2θ)]^-1/4^
0 constraints	Extinction coefficient: 0.047 (4)
Primary atom site location: structure-invariant direct methods	

### Fractional atomic coordinates and isotropic or equivalent isotropic displacement parameters (Å^2^)

**Table:**

	*x*	*y*	*z*	*U*_iso_*/*U*_eq_	
C1	0.90505 (16)	0.73779 (15)	0.01317 (13)	0.0338 (3)	
C2	0.99879 (16)	0.69360 (14)	0.11020 (13)	0.0332 (3)	
C3	0.96024 (16)	0.65343 (14)	0.22245 (13)	0.0331 (3)	
H3	0.9429	0.5490	0.2121	0.040*	
C4	0.79214 (17)	0.65768 (15)	0.19639 (13)	0.0350 (3)	
C5	0.70197 (17)	0.70668 (15)	0.09468 (13)	0.0345 (3)	
C6	0.4686 (2)	0.7677 (2)	−0.04503 (17)	0.0510 (4)	
H6A	0.3650	0.7650	−0.0459	0.076*	
H6B	0.4388	0.7028	−0.1331	0.076*	
H6C	0.5440	0.8691	−0.0293	0.076*	
C11	0.92473 (17)	0.76501 (15)	−0.10669 (14)	0.0359 (3)	
C12	1.0837 (2)	0.85749 (18)	−0.08937 (16)	0.0461 (4)	
H12	1.1788	0.9101	−0.0002	0.055*	
C13	1.1012 (2)	0.8717 (2)	−0.20466 (17)	0.0538 (4)	
H13	1.2085	0.9336	−0.1929	0.065*	
C14	0.9618 (2)	0.79536 (19)	−0.33610 (17)	0.0535 (4)	
H14	0.9749	0.8041	−0.4134	0.064*	
C15	0.8026 (2)	0.7059 (2)	−0.35402 (16)	0.0561 (4)	
H15	0.7076	0.6555	−0.4434	0.067*	
C16	0.7828 (2)	0.69049 (19)	−0.23976 (15)	0.0479 (4)	
H16	0.6746	0.6303	−0.2521	0.057*	
C21	1.13736 (18)	0.66852 (16)	0.10462 (14)	0.0384 (3)	
C31	1.11327 (17)	0.75233 (15)	0.36706 (13)	0.0352 (3)	
C32	1.1702 (2)	0.90599 (18)	0.41307 (17)	0.0535 (4)	
H32	1.1129	0.9507	0.3556	0.064*	
C33	1.3112 (3)	0.9951 (2)	0.54360 (19)	0.0682 (5)	
H33	1.3490	1.0989	0.5750	0.082*	
C34	1.3930 (2)	0.9269 (2)	0.62448 (17)	0.0636 (5)	
C35	1.3422 (2)	0.7764 (2)	0.58302 (18)	0.0657 (5)	
H35	1.4015	0.7332	0.6409	0.079*	
C36	1.2002 (2)	0.68821 (19)	0.45290 (16)	0.0515 (4)	
H36	1.1630	0.5843	0.4230	0.062*	
N1	0.72180 (15)	0.59726 (14)	0.27462 (12)	0.0421 (3)	
N2	0.55637 (15)	0.71659 (15)	0.06560 (12)	0.0433 (3)	
H2	0.5096	0.6909	0.1159	0.052*	
N3	1.24589 (17)	0.64228 (17)	0.10373 (16)	0.0549 (4)	
O1	0.76236 (12)	0.75260 (11)	0.00900 (10)	0.0393 (2)	
O2	0.58447 (15)	0.60002 (16)	0.26385 (13)	0.0625 (3)	
O3	0.79677 (15)	0.53945 (13)	0.35412 (11)	0.0523 (3)	
F	1.53011 (18)	1.01296 (16)	0.75406 (12)	0.1082 (5)	

### Atomic displacement parameters (Å^2^)

**Table:**

	*U*^11^	*U*^22^	*U*^33^	*U*^12^	*U*^13^	*U*^23^
C1	0.0310 (6)	0.0395 (7)	0.0353 (7)	0.0147 (5)	0.0211 (6)	0.0115 (5)
C2	0.0314 (6)	0.0383 (7)	0.0328 (6)	0.0150 (5)	0.0190 (5)	0.0104 (5)
C3	0.0350 (7)	0.0355 (6)	0.0338 (6)	0.0166 (5)	0.0197 (6)	0.0137 (5)
C4	0.0338 (7)	0.0421 (7)	0.0334 (6)	0.0147 (6)	0.0212 (6)	0.0140 (5)
C5	0.0318 (7)	0.0405 (7)	0.0341 (6)	0.0142 (6)	0.0205 (5)	0.0112 (5)
C6	0.0396 (8)	0.0696 (10)	0.0509 (9)	0.0300 (8)	0.0219 (7)	0.0255 (8)
C11	0.0388 (7)	0.0423 (7)	0.0354 (7)	0.0199 (6)	0.0231 (6)	0.0163 (6)
C12	0.0416 (8)	0.0537 (9)	0.0415 (8)	0.0146 (7)	0.0239 (7)	0.0173 (7)
C13	0.0538 (9)	0.0618 (10)	0.0579 (10)	0.0201 (8)	0.0396 (8)	0.0275 (8)
C14	0.0731 (11)	0.0616 (10)	0.0465 (9)	0.0319 (9)	0.0425 (9)	0.0265 (8)
C15	0.0614 (10)	0.0669 (11)	0.0342 (8)	0.0229 (8)	0.0225 (7)	0.0167 (7)
C16	0.0410 (8)	0.0590 (9)	0.0407 (8)	0.0158 (7)	0.0218 (7)	0.0186 (7)
C21	0.0354 (7)	0.0435 (7)	0.0385 (7)	0.0164 (6)	0.0208 (6)	0.0139 (6)
C31	0.0355 (7)	0.0410 (7)	0.0337 (7)	0.0170 (6)	0.0201 (6)	0.0148 (5)
C32	0.0596 (10)	0.0441 (8)	0.0478 (9)	0.0218 (7)	0.0186 (8)	0.0168 (7)
C33	0.0755 (13)	0.0427 (9)	0.0531 (10)	0.0088 (9)	0.0198 (9)	0.0062 (8)
C34	0.0530 (10)	0.0673 (11)	0.0362 (8)	0.0059 (9)	0.0102 (7)	0.0117 (8)
C35	0.0593 (11)	0.0757 (12)	0.0454 (9)	0.0271 (9)	0.0106 (8)	0.0284 (9)
C36	0.0544 (9)	0.0491 (9)	0.0445 (8)	0.0236 (7)	0.0164 (7)	0.0198 (7)
N1	0.0393 (7)	0.0504 (7)	0.0388 (6)	0.0150 (5)	0.0241 (5)	0.0170 (5)
N2	0.0362 (6)	0.0633 (8)	0.0435 (7)	0.0258 (6)	0.0260 (5)	0.0237 (6)
N3	0.0446 (8)	0.0647 (9)	0.0710 (9)	0.0295 (7)	0.0370 (7)	0.0240 (7)
O1	0.0381 (5)	0.0574 (6)	0.0411 (5)	0.0273 (5)	0.0273 (4)	0.0259 (5)
O2	0.0495 (7)	0.0981 (9)	0.0680 (8)	0.0353 (7)	0.0449 (6)	0.0453 (7)
O3	0.0570 (7)	0.0622 (7)	0.0492 (6)	0.0258 (6)	0.0314 (5)	0.0328 (5)
F	0.0874 (9)	0.0959 (10)	0.0492 (7)	−0.0025 (7)	−0.0066 (6)	0.0070 (6)

### Geometric parameters (Å, º)

**Table:**

C1—C2	1.3293 (18)	C13—H13	0.9300
C1—O1	1.3795 (15)	C14—C15	1.372 (2)
C1—C11	1.4717 (17)	C14—H14	0.9300
C2—C21	1.4276 (18)	C15—C16	1.382 (2)
C2—C3	1.5089 (17)	C15—H15	0.9300
C3—C4	1.5012 (18)	C16—H16	0.9300
C3—C31	1.5222 (18)	C21—N3	1.1369 (18)
C3—H3	0.9800	C31—C32	1.374 (2)
C4—C5	1.3796 (19)	C31—C36	1.374 (2)
C4—N1	1.3855 (17)	C32—C33	1.382 (2)
C5—N2	1.3130 (17)	C32—H32	0.9300
C5—O1	1.3566 (15)	C33—C34	1.354 (3)
C6—N2	1.4498 (19)	C33—H33	0.9300
C6—H6A	0.9600	C34—C35	1.351 (3)
C6—H6B	0.9600	C34—F	1.3599 (19)
C6—H6C	0.9600	C35—C36	1.381 (2)
C11—C12	1.382 (2)	C35—H35	0.9300
C11—C16	1.386 (2)	C36—H36	0.9300
C12—C13	1.380 (2)	N1—O3	1.2375 (16)
C12—H12	0.9300	N1—O2	1.2558 (16)
C13—C14	1.368 (2)	N2—H2	0.8600
			
C2—C1—O1	121.49 (11)	C13—C14—H14	120.0
C2—C1—C11	127.43 (12)	C15—C14—H14	120.0
O1—C1—C11	110.91 (11)	C14—C15—C16	120.27 (15)
C1—C2—C21	119.74 (12)	C14—C15—H15	119.9
C1—C2—C3	124.43 (11)	C16—C15—H15	119.9
C21—C2—C3	115.60 (11)	C15—C16—C11	119.75 (14)
C4—C3—C2	108.51 (10)	C15—C16—H16	120.1
C4—C3—C31	114.77 (10)	C11—C16—H16	120.1
C2—C3—C31	111.06 (10)	N3—C21—C2	175.98 (15)
C4—C3—H3	107.4	C32—C31—C36	118.79 (14)
C2—C3—H3	107.4	C32—C31—C3	121.42 (12)
C31—C3—H3	107.4	C36—C31—C3	119.77 (12)
C5—C4—N1	120.05 (12)	C31—C32—C33	120.93 (15)
C5—C4—C3	123.99 (11)	C31—C32—H32	119.5
N1—C4—C3	115.78 (11)	C33—C32—H32	119.5
N2—C5—O1	111.37 (11)	C34—C33—C32	118.19 (16)
N2—C5—C4	128.35 (12)	C34—C33—H33	120.9
O1—C5—C4	120.27 (11)	C32—C33—H33	120.9
N2—C6—H6A	109.5	C35—C34—C33	122.83 (16)
N2—C6—H6B	109.5	C35—C34—F	118.40 (17)
H6A—C6—H6B	109.5	C33—C34—F	118.76 (18)
N2—C6—H6C	109.5	C34—C35—C36	118.57 (16)
H6A—C6—H6C	109.5	C34—C35—H35	120.7
H6B—C6—H6C	109.5	C36—C35—H35	120.7
C12—C11—C16	119.61 (13)	C31—C36—C35	120.69 (15)
C12—C11—C1	121.15 (12)	C31—C36—H36	119.7
C16—C11—C1	119.16 (12)	C35—C36—H36	119.7
C13—C12—C11	119.86 (14)	O3—N1—O2	121.01 (11)
C13—C12—H12	120.1	O3—N1—C4	118.35 (12)
C11—C12—H12	120.1	O2—N1—C4	120.64 (12)
C14—C13—C12	120.41 (15)	C5—N2—C6	124.79 (12)
C14—C13—H13	119.8	C5—N2—H2	117.6
C12—C13—H13	119.8	C6—N2—H2	117.6
C13—C14—C15	120.07 (14)	C5—O1—C1	120.68 (10)
			
O1—C1—C2—C21	175.21 (12)	C1—C11—C16—C15	−175.02 (14)
C11—C1—C2—C21	0.5 (2)	C1—C2—C21—N3	−149 (2)
O1—C1—C2—C3	0.9 (2)	C3—C2—C21—N3	25 (2)
C11—C1—C2—C3	−173.81 (12)	C4—C3—C31—C32	−60.76 (17)
C1—C2—C3—C4	5.36 (18)	C2—C3—C31—C32	62.76 (17)
C21—C2—C3—C4	−169.14 (11)	C4—C3—C31—C36	120.98 (14)
C1—C2—C3—C31	−121.67 (14)	C2—C3—C31—C36	−115.50 (14)
C21—C2—C3—C31	63.83 (14)	C36—C31—C32—C33	−0.5 (3)
C2—C3—C4—C5	−6.35 (18)	C3—C31—C32—C33	−178.80 (15)
C31—C3—C4—C5	118.52 (14)	C31—C32—C33—C34	0.6 (3)
C2—C3—C4—N1	168.72 (11)	C32—C33—C34—C35	−0.2 (3)
C31—C3—C4—N1	−66.41 (15)	C32—C33—C34—F	−178.89 (18)
N1—C4—C5—N2	6.2 (2)	C33—C34—C35—C36	−0.4 (3)
C3—C4—C5—N2	−178.88 (13)	F—C34—C35—C36	178.36 (17)
N1—C4—C5—O1	−173.76 (12)	C32—C31—C36—C35	0.0 (2)
C3—C4—C5—O1	1.1 (2)	C3—C31—C36—C35	178.28 (15)
C2—C1—C11—C12	−51.4 (2)	C34—C35—C36—C31	0.5 (3)
O1—C1—C11—C12	133.39 (14)	C5—C4—N1—O3	172.24 (12)
C2—C1—C11—C16	125.37 (16)	C3—C4—N1—O3	−3.05 (18)
O1—C1—C11—C16	−49.82 (17)	C5—C4—N1—O2	−7.1 (2)
C16—C11—C12—C13	−1.8 (2)	C3—C4—N1—O2	177.62 (12)
C1—C11—C12—C13	174.97 (14)	O1—C5—N2—C6	0.9 (2)
C11—C12—C13—C14	0.3 (3)	C4—C5—N2—C6	−179.11 (14)
C12—C13—C14—C15	1.1 (3)	N2—C5—O1—C1	−173.84 (11)
C13—C14—C15—C16	−1.1 (3)	C4—C5—O1—C1	6.16 (19)
C14—C15—C16—C11	−0.4 (3)	C2—C1—O1—C5	−7.26 (19)
C12—C11—C16—C15	1.8 (2)	C11—C1—O1—C5	168.27 (11)

### Hydrogen-bond geometry (Å, º)

**Table:**

*D*—H···*A*	*D*—H	H···*A*	*D*···*A*	*D*—H···*A*
N2—H2···O2	0.86	1.99	2.6089 (16)	128
N2—H2···N3^i^	0.86	2.30	2.9811 (17)	136
C6—H6*A*···N3^i^	0.96	2.60	3.222 (2)	123

Symmetry code: (i) *x*−1, *y*, *z*.
